# Precision-cut kidney slices (PCKS) to study development of renal fibrosis and efficacy of drug targeting *ex vivo*

**DOI:** 10.1242/dmm.020172

**Published:** 2015-10-01

**Authors:** Fariba Poosti, Bao Tung Pham, Dorenda Oosterhuis, Klaas Poelstra, Harry van Goor, Peter Olinga, Jan-Luuk Hillebrands

**Affiliations:** 1 Departments of Pathology and Medical Biology, Division of Pathology, University Medical Center Groningen, University of Groningen, Groningen, 9713 GZ, The Netherlands; 2 Department of Pharmaceutical Technology and Biopharmacy, University of Groningen, Groningen, 9713 AV, The Netherlands; 3 Department of Pharmacokinetics, Toxicology and Targeting, University of Groningen, Groningen, 9713 AV, The Netherlands

**Keywords:** Drug targeting, Fibrosis, Kidney, Tissue slices

## Abstract

Renal fibrosis is a serious clinical problem resulting in the greatest need for renal replacement therapy. No adequate preventive or curative therapy is available that could be clinically used to target renal fibrosis specifically. The search for new efficacious treatment strategies is therefore warranted. Although *in vitro* models using homogeneous cell populations have contributed to the understanding of the pathogenetic mechanisms involved in renal fibrosis, these models poorly mimic the complex *in vivo* milieu. Therefore, we here evaluated a precision-cut kidney slice (PCKS) model as a new, multicellular *ex vivo* model to study the development of fibrosis and its prevention using anti-fibrotic compounds. Precision-cut slices (200-300 μm thickness) were prepared from healthy C57BL/6 mouse kidneys using a Krumdieck tissue slicer. To induce changes mimicking the fibrotic process, slices were incubated with TGFβ1 (5 ng/ml) for 48 h in the presence or absence of the anti-fibrotic cytokine IFNγ (1 µg/ml) or an IFNγ conjugate targeted to PDGFRβ (PPB-PEG-IFNγ). Following culture, tissue viability (ATP-content) and expression of α-SMA, fibronectin, collagen I and collagen III were determined using real-time PCR and immunohistochemistry. Slices remained viable up to 72 h of incubation, and no significant effects of TGFβ1 and IFNγ on viability were observed. TGFβ1 markedly increased α-SMA, fibronectin and collagen I mRNA and protein expression levels. IFNγ and PPB-PEG-IFNγ significantly reduced TGFβ1-induced fibronectin, collagen I and collagen III mRNA expression, which was confirmed by immunohistochemistry. The PKCS model is a novel tool to test the pathophysiology of fibrosis and to screen the efficacy of anti-fibrotic drugs *ex vivo* in a multicellular and pro-fibrotic milieu. A major advantage of the slice model is that it can be used not only for animal but also for (fibrotic) human kidney tissue.

## Abstract (precis)

**Drug Discovery Collection:** TGFβ induces renal fibrosis in *ex vivo* cultured precision-cut kidney slices, which can be attenuated by IFNγ.

## INTRODUCTION

Renal fibrosis is a major contributor to the development of chronic kidney disease (CKD) and is characterized by accumulation of myofibroblasts and excessive extracellular matrix deposition (Eddy, 2014; Friedman et al., 2013). CKD culminates in end-stage renal disease (ESRD), resulting in significant morbidity and mortality, for which the only available therapy is dialysis or renal transplantation (Levey et al., 2007). The pathophysiology of renal fibrosis is not fully understood, and currently no adequate preventive or curative therapy is clinically available (Boor et al., 2010). Research focusing on unraveling the pathophysiology of renal fibrosis is therefore warranted. Traditionally, renal research is performed in *in vitro* cell culture systems or small rodent models as well as on human renal biopsies or explants (Westra et al., 2013). Despite the fact that *in vitro* experiments using homogeneous single cell preparations allow functional analyses of a specific cell type, the major disadvantage is a lack of cell heterogeneity and cellular microarchitecture; phenomena that are undoubtedly involved in determining and driving the fibrotic process (Vickers and Fisher, 2005; Westra et al., 2013). Because of ethical constraints, the use of animal models is increasingly discouraged. Furthermore, use of human tissue biopsies is by definition descriptive in nature and prohibits functional studies.

The availability of an *ex vivo* model system that does allow functional analyses on the development of renal fibrosis is therefore highly desired. Recently, precision-cut tissue slices (PCTS) have been increasingly used to study the development of liver fibrosis (Hadi et al., 2013; Westra et al., 2014a,b). In this model, cell-cell and cell-extracellular matrix (ECM) interactions are preserved. As yet, it is unknown whether precision-cut kidney slices (PCKS) can be used to study the development of renal fibrosis and to screen the efficacy of anti-fibrotic compounds, e.g. IFNγ. IFNγ is a pleiotropic cytokine produced by various activated immune cells, including NK cells and T cells (Farrar and Schreiber, 1993). In addition to its pro-inflammatory effects, IFNγ has prominent anti-fibrotic effects. IFNγ is able to inhibit fibroblast activation and proliferation, and also reduces ECM synthesis (Lee et al., 2013; Oldroyd et al., 1999; Strutz et al., 2000; Weng et al., 2005; Yao et al., 2011). These properties make IFNγ a potential molecule for therapeutic use to target fibrosis. However, the short half-life and undesirable systemic side-effects clearly limit clinical utility of IFNγ as an anti-fibrotic drug (Cleland and Jones, 1996; Gu et al., 2005; King et al., 2009). To overcome these problems, we employed a drug-targeting strategy in which IFNγ was targeted specifically to activated myofibroblasts in animal models of liver and kidney fibrosis, using PDGFRβ as docking receptor for the PPB-PEG-IFNγ conjugate (PPB: PDGFRβ-recognizing receptor) (Bansal et al., 2011a,b,c, 2012; Poosti et al., 2015).

The aim of the present study was to validate the PCKS model for the development of TGFβ1-induced renal fibrosis and to evaluate the potential anti-fibrotic effects of IFNγ and PPB-PEG-IFNγ in this *ex vivo* model.

**TRANSLATIONAL IMPACT**

**Clinical issue**

Renal fibrosis is a severe clinical problem and is the most common pathogenetic cause of chronic kidney disease (CKD). It is characterized by excessive formation of scar tissue due to extracellular matrix (ECM) deposition and myofibroblast activation. As yet, effective treatment is still lacking. Understanding the pathophysiology of renal fibrosis is required for the development of new efficacious treatment strategies. *In vitro* models using homogeneous cell populations have contributed to the understanding of the mechanisms involved in fibrosis but they mimic its complex *in vivo* milieu only poorly. Precision-cut tissue slices (PCTS), a three-dimensional multicellular environment, are a powerful system to provide insight into mechanisms of organ injury. Tissue slices have the biologically relevant structural features of *in vivo* tissues. The aim of this study was to validate the precision-cut kidney slice (PCKS) model for the development of renal fibrosis and to evaluate the potential anti-fibrotic effects of IFNγ and an IFNγ conjugate in this *ex vivo* model.

**Results**

In this study, PCKSs were prepared from healthy C57BL/6 mouse kidneys using a Krumdieck tissue slicer. To induce changes that mimic the fibrotic process, slices were incubated with TGFβ1 for 48 h in the presence or absence of the anti-fibrotic cytokine IFNγ or an IFNγ conjugate (PPB-PEG-IFNγ). After culture preparation, tissue viability (measured as ATP-content) and expression of fibrosis markers were determined using real-time PCR and immunohistochemistry. Slices remained viable up to 72 h of incubation and no significant effects of TGFβ1 and IFNγ on viability were observed. TGFβ1 markedly increased alpha-smooth muscle actin (α-SMA; a marker of myofibroblast formation) and the ECM proteins fibronectin and collagen I at both mRNA and protein levels. IFNγ and PPB-PEG-IFNγ significantly reduced TGFβ1-induced fibronectin and collagen I mRNA expression, which was confirmed by immunohistochemistry.

**Implications and future directions**

The PCKS model described here is a novel tool to test the pathophysiology of early onset fibrosis and to screen the efficacy of anti-fibrotic drugs *ex vivo* in a multicellular and pro-fibrotic milieu. The PCKS model might help to bridge the gap between *in vitro* cell culture systems and human *in vivo* studies. Importantly, the use of kidney tissue slices also contributes to the implementation of the ‘3R’ principles, as it can replace and reduce animal experiments. Future studies need to be performed using human PCKS to validate the ‘treatment-to-target-fibrosis’ approach described in this study. These preclinical studies should then pave the road towards clinical studies on treatments for renal fibrosis.

## RESULTS

### Viability of mouse kidney slices (mPCKS) during incubation

**Fig. 1.**
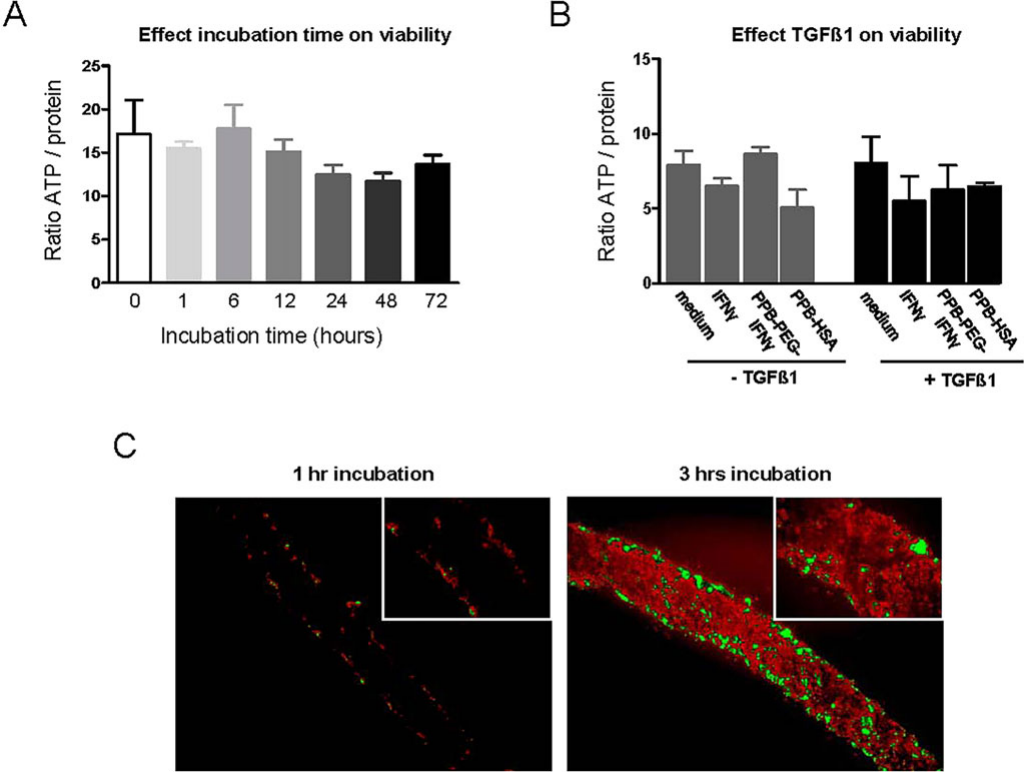
**Effect of incubation time and conditions on mPCKS viability, and uptake of FITC-lysozyme by mPCKS.** (A) Non-cultured normal kidney slices (*n*=3) directly after the slicing procedure (0) and after different incubation intervals. (B) Effect of 48 h of incubation with TGFβ1 on ATP content of mPCKS in the presence of IFNγ, PPB-PEG-IFNγ and PPB-HSA with or without TGFβ1. (C) mPCKS were incubated with FITC-lysozyme (model protein) for 1 h (left panel) and 3 h (right panel) to determine penetration of exogenously added protein in mPCKS (magnification: 100×, inset: 400×).

In order to determine viability of mPCKS, ATP levels per mg of protein were assessed directly after preparation of slices, and after 1, 6, 12, 24, 48 and 72 h of incubation (Fig. 1A). Kidney slices remained viable up to 72 h of incubation; however, a minor, non-significant decrease in ATP levels was observed after 24 h of incubation. Up to 72 h slices retained constant ATP levels. As the morphology of kidney slices started to deteriorate after 72 h of incubation (based on PAS staining, data not shown), the experiments with TGFβ1 and anti-fibrotic compounds were conducted with a maximal culture time of 48 h. In a separate experiment, we first examined the viability of slices after 48 h of incubation with or without TGFβ1 in the presence of free IFNγ, PPB-PEG-IFNγ or PPB-HSA. As shown in Fig. 1B, TGFβ1 did not significantly alter ATP content. Furthermore, free IFNγ, PPB-PEG-IFNγ or PPB-HSA did not affect ATP levels independent of presence or absence of TGFβ1.

### Uptake of FITC-lysozyme by mPCKS

We next analyzed whether exogenously added proteins can reach the center of mPCKS during incubation. We used FITC-conjugated lysozyme as model protein. As shown in Fig. 1C, after 1 h of incubation, FITC-conjugated lysozyme was only present at the outer surface area of mPCKS. However, after 3 h of incubation, fluorescence was detected throughout the thickness of the slices, with high staining intensity (depicted in green) also observed within the center of the slice.

### Biological activity of IFNγ and PPB-PEG-IFNγ *ex vivo*

**Fig. 2.**
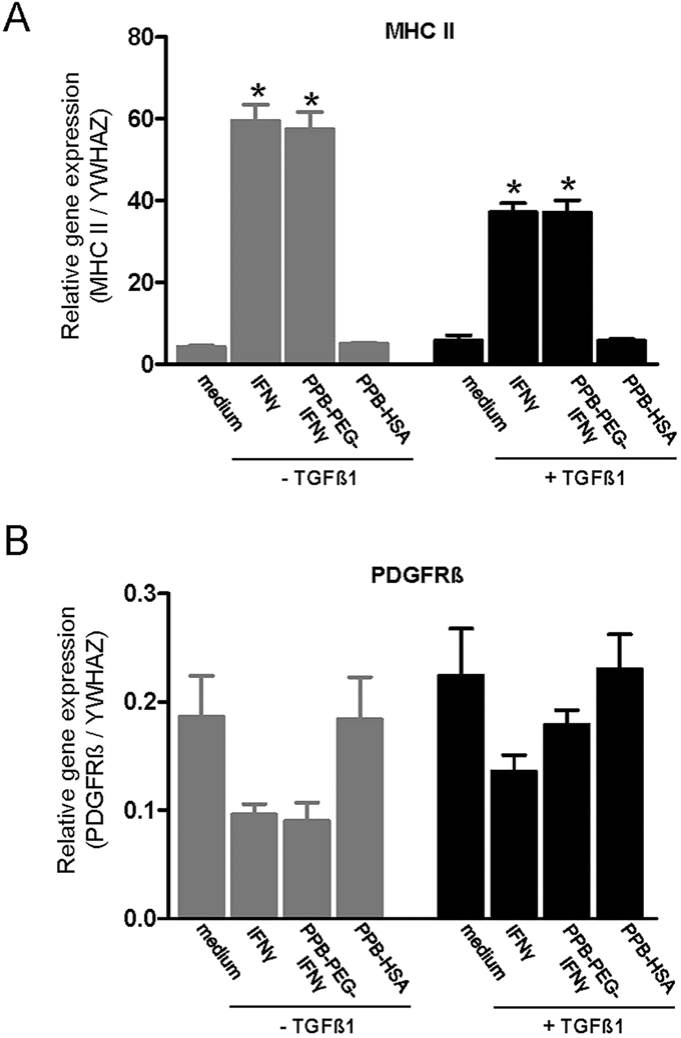
**MHC II and PDGFRβ expression in mPCKS.** (A) mRNA expression of MHC II in pre-fibrotic (+TGFβ1) and non-fibrotic (−TGFβ1) kidney slices treated with IFNγ, PPB-PEG-IFNγ or PPB-HSA. (B) mRNA expression of PDGFRβ in pre-fibrotic (+TGFβ1) and non-fibrotic (−TGFβ1) kidney slices treated with IFNγ, PPB-PEG-IFNγ or PPB-HSA. *P<0.05 versus medium.

To determine the biological activity of free IFNγ and PPB-PEG-IFNγ, we assessed major histocompatibility class II (MHC II) expression, which is known to be upregulated by IFNγ (Bansal et al., 2011a). Expression of MHC II in response to IFNγ was analyzed in TGFβ- and non-TGFβ-incubated slices. qRT-PCR results showed that incubation of kidney slices in the presence of both free IFNγ and PPB-PEG-IFNγ caused a significant upregulation of MHC II expression compared with medium, effects that were not influenced by TGFβ1 (Fig. 2A). However, PPB-HSA (the PDGFRβ-specific drug delivery carrier without IFNγ) did not induce MHC II expression. These data indicate that IFNγ and PPB-PEG-IFNγ retained their biological activity in kidney slices.

### PDGFRβ expression in mPCKS

We recently showed increased interstitial PDGFRβ expression in both human and mouse fibrotic renal tissue which co-localized with α-SMA^+^ interstitial myofibroblasts (Poosti et al., 2015). High expression of PDGFRβ on interstitial myofibroblasts indicates that this receptor is an appropriate target for fibroblast-specific delivery. Therefore, we next analyzed whether cultured mPCKS in the presence of TGFβ1 are also characterized by PDGFRβ expression. As shown in Fig. 2B, mPCKS cultured with medium but without TGFβ1 clearly expressed PDGFRβ, which was not increased by TGFβ1. A clear, but non-significant reduction in PDGFRβ expression was observed after incubation in the presence of free IFNγ and PPB-PEG-IFNγ (Fig. 2B).

### Induction of α-SMA and ECM expression in mPCKS by TGFβ1

**Fig. 3.**
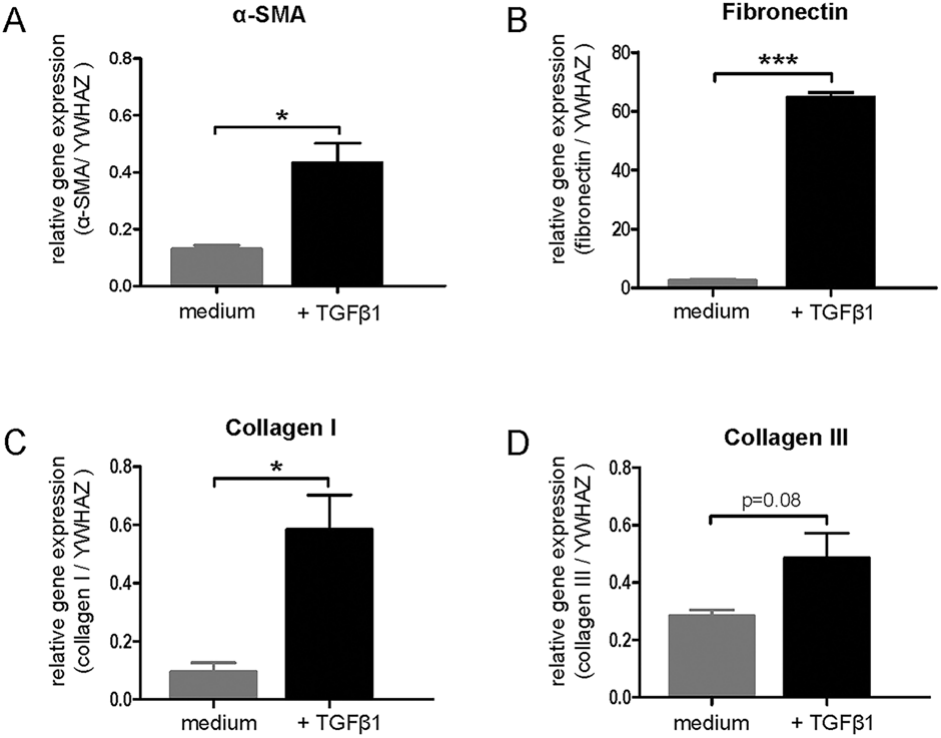
**TGFβ1 (5 ng/ml) induces expression of fibrotic markers in mPCKS.** mRNA expression of (A) α-SMA, (B) fibronectin, (C) collagen I and (D) collagen III in mouse kidney slices after 48 h of incubation. *P<0.05, ***P<0.001 versus medium.

**Fig. 4.**
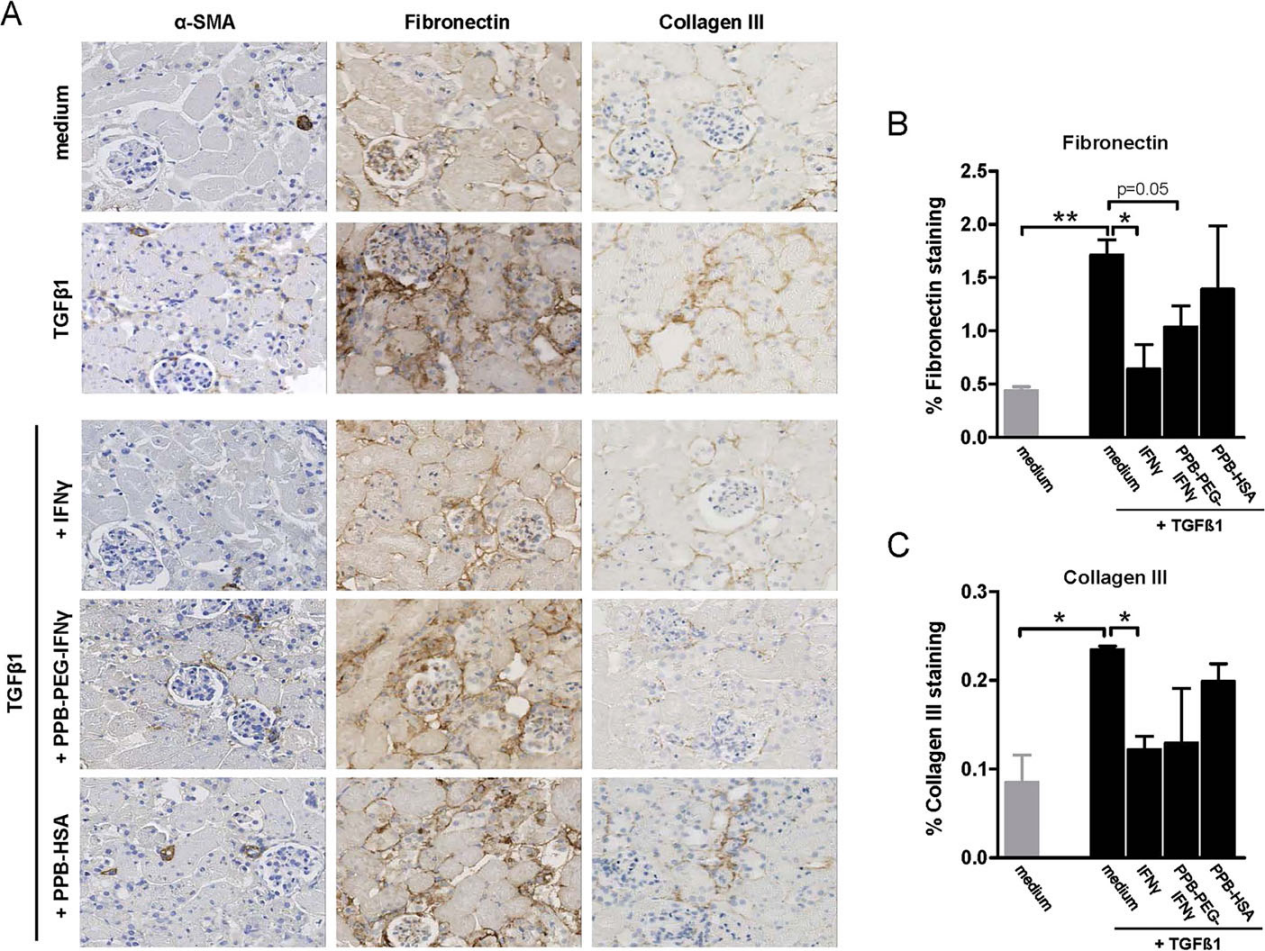
**Protein expression of α-SMA, fibronectin and collagen III in mPCKS cultured without TGFβ (medium), or with TGFβ in the presence of free IFNγ, PPB-PEG-IFNγ or PPB-HSA.** (A) Photomicrographs of immunohistochemistry for α-SMA, fibronectin and collagen III on mPCKS after 48 h of culture (magnification: 200×). Quantitative analysis of fibronectin (B) and collagen III (C) expression in mPCKS cultured under various conditions. *P<0.05, **P<0.01; Student's *t*-test.

To study development of (pre)fibrosis in mPCKS in the presence of TGFβ1, mRNA expression of fibrosis markers was determined after 48 h of incubation with TGFβ1 (5 ng/ml). As shown in Fig. 3, we observed significant upregulation of α-SMA (A), fibronectin (B) and collagen I (C) mRNA expression upon incubation with TGFβ1 compared with non-TGFβ1-incubated control (medium) slices. TGFβ1 also increased expression of collagen III; however, without reaching the level of statistical significance (*P*=0.08). We next tested by immunohistochemistry whether increased mRNA expression of fibrosis markers also translated into altered protein expression levels. As shown in Fig. 4A (two upper rows), incubation with TGFβ1 indeed markedly increased α-SMA, fibronectin and collagen III expression.

### Effect of free IFNγ and PPB-PEG-IFNγ on the expression of fibrosis markers

**Fig. 5.**
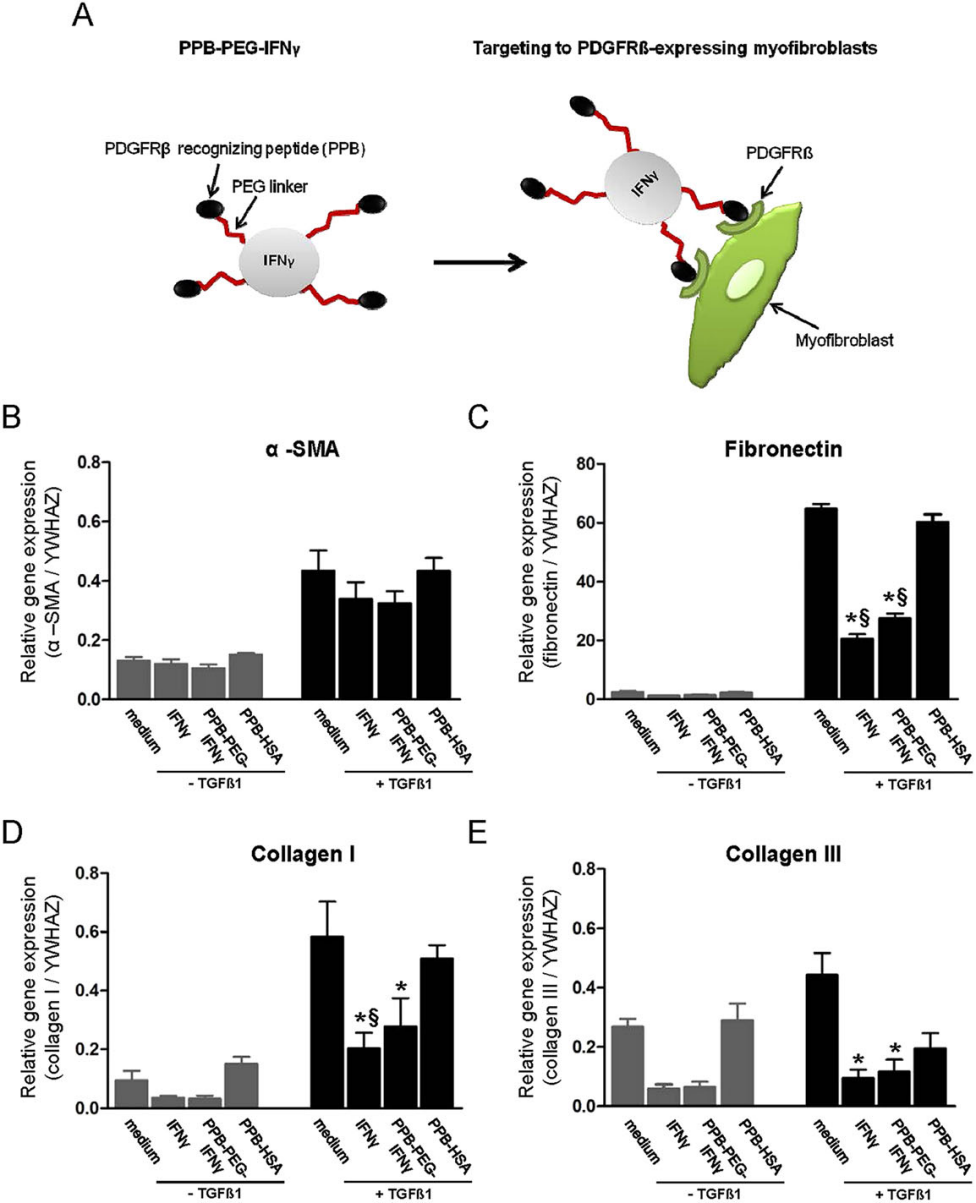
**Effect of IFNγ and PPB-PEG-IFNγ on the expression of fibrotic markers in mPCKS.** (A) Schematic of targeted delivery of IFNγ to PDGFRβ-expressing myofibroblasts in mPCKS. Relative gene expression of (B) α-SMA, (C) collagen I, (D) fibronectin and (E) collagen III. *P<0.05 versus medium; ^§^*P*<0.05 versus PPB-HSA.

To investigate whether free IFNγ and PPB-PEG-IFNγ treatment ameliorates fibrosis, qRT-PCR analysis was performed on fibrotic and healthy control slices. As already shown in Fig. 3, TGFβ1 induced α-SMA, fibronectin, collagen I and collagen III mRNA expression. Fig. 5A depicts a schematic representation of the targeting strategy in which IFNγ is targeted towards PDGFRβ-expressing myofibroblasts using the PPB-PEG-IFNγ conjugate. In cultured slices incubated with TGFβ plus either IFNγ or PPB-PEG-IFNγ, mRNA expression levels for fibronectin, collagen I and collagen III were significantly reduced compared with slices incubated with TGFβ alone (Fig. 5C-E). IFNγ and PPB-PEG-IFNγ also showed somewhat reduced α-SMA expression (Fig. 5B); however, without reaching the level of statistical significance. To determine whether IFNγ, PPB-PEG-IFNγ and PPB-HSA have protective effects in spontaneous induction of fibrosis, kidney slices were also incubated with the aforementioned compounds in the absence of TGFβ1. In this condition, we did not observe significant reduction of fibrosis maker expression in the presence of free IFNγ or PPB-PEG-IFNγ, although clear trends were observed for collagen I (Fig. 5D) and collagen III (Fig. 5E).

To determine whether mRNA expression profiles also translated into differential protein expression levels, immunohistochemistry for α-SMA, fibronectin and collagen III was performed. Staining for collagen I was technically not feasible. Representative photomicrographs of α-SMA, fibronectin and collagen III staining are shown in Fig. 4A. Microscopic analysis revealed no clear differences in α-SMA expression (similar to mRNA expression, Fig. 3B). Quantitative analysis for fibronectin (Fig. 4B) showed significantly (*P*<0.01) increased expression levels in slices cultured in the presence of TGFβ1 (compared with medium), which was reduced by IFNγ (*P*<0.05) and PPB-PEG-IFNγ (*P*=0.05). Similar results were observed for the expression of collagen III (Fig. 4C). PPB-HSA did not show any significant inhibitory effect on neither mRNA nor protein levels.

## DISCUSSION

The present study reveals the applicability of PCKS as: (1) an *ex vivo* model to study renal fibrogenesis, and (2) to test anti-fibrotic effects of IFNγ and targeted IFNγ aiming at reducing renal fibrosis. Specifically, the results demonstrate that incubation of mPCKS with TGFβ1 resulted in upregulation of fibronectin, collagen I and α-SMA expression, which indicates that mPCKS represent an ideal model to study the onset of early fibrosis. Preserved expression of α-SMA suggests that fibroblasts remained active during culture. Still, intervention with free IFNγ and PPB-PEG-IFNγ dampened TGFβ1-induced expression of fibronectin, collagen I and collagen III, thereby demonstrating the anti-fibrotic potential of both free and targeted IFNγ.

Renal fibrosis results from excessive ECM accumulation and fibroblast proliferation (Duffield, 2014; LeBleu et al., 2013; Strutz and Zeisberg, 2006). In order to develop efficacious anti-fibrotic therapy detailed knowledge of the underlying pathophysiology is necessary. It has long been recognized that *in vitro* studies can provide useful information on the mechanisms of disease (Westra et al., 2013, 2014a). PCTS, a three-dimensional multicellular environment, is a powerful model to provide insight into mechanisms of organ injury (Basile et al., 2012; Flamant et al., 2006; Nagae et al., 2008; Tharaux et al., 2000; van Swelm et al., 2014). Tissue slices have the biologically relevant structural features of *in vivo* tissues (de Kanter et al., 2002). PCTS have been widely used as *ex vivo* model to study the development of liver and intestinal fibrosis (Westra et al., 2013). *Ex vivo* liver and intestinal slices maintain cell heterogeneity and tissue architecture, providing an appropriate tool to study multicellular processes such as fibrosis (de Kanter et al., 2005; van de Kerkhof et al., 2005; Westra et al., 2014b; de Graaf et al., 2010).

In this study, mouse kidney slices remained viable during the incubation period of 72 h, as determined by ATP content, a parameter generally used as indicator of viability. In addition, the increased expression of fibrosis markers in response to TGFβ1 also indicated that the relevant cells involved in fibrogenesis (i.e. fibroblasts) remained viable. It is generally accepted that stimulation with TGFβ1 induces differentiation of local fibroblasts into myofibroblasts which start to produce and secrete ECM. In line with this, we recently demonstrated that the same concentration of TGFβ (5 ng/ml) used in this study induces collagen and α-SMA expression in NIH3T3 fibroblasts, indicating TGFβ1-induced fibroblast-to-myofibroblast differentiation (Poosti et al., 2015). Although other renal cell types (including tubular epithelial cells, endothelial cells, podocytes, mesangial cells and pericytes) may also respond to TGFβ1 (Meng et al., 2015), our immunohistochemistry data support tubulointerstitial (myo)fibroblasts as the main responders to TGFβ1 in mPCKS. Activation and/or proliferation of fibrogenic cells, which was accompanied by increased production of ECM, indicates that kidney slices could be a useful tool to study the effects of anti-fibrotic compounds in a multicellular environment.

IFNγ could be a useful therapeutic target to attenuate the development of renal fibrosis. Despite its potential effectiveness, application of IFNγ in clinical trials resulted in negative data (Cleland and Jones, 1996; Gu et al., 2005; King et al., 2009). The main reasons for the lack of clinical effects are the poor pharmacokinetic profile of IFNγ and the severe side effects due to ubiquitous expression of IFNγ receptors. Therefore, targeted delivery of IFNγ to key cells is thought to be a prerequisite to enhance its therapeutic efficacy and at the same time reduce systemic side effects. Recently, we studied anti-fibrotic effects of IFNγ and PEGylated IFNγ (PPB-PEG-IFNγ) targeted to PDGFRβ-expressing myofibroblasts in an animal model of renal fibrosis (Poosti et al., 2015). Our strategy was to conjugate the cyclic PDGFRβ-binding peptide (PPB) to mouse IFNγ via a PEG linker (PPB-PEG-IFNγ) to achieve specific delivery of IFNγ to PDGFRβ-expressing cells, due to upregulation of this receptor in fibrotic diseases. In order to assess the applicability of the PCKS model for testing anti-fibrotic compounds, we examined IFNγ and targeted IFNγ (PPB-PEG-IFNγ) on TGFβ-activated pre-fibrotic slices and on non-activated slices. Contrary to our expectation, PDGFRβ expression was not affected by TGFβ1. This might be explained by the fact that fibrosis is a multifactorial process, and induction of PDGFRβ expression *in vivo* might therefore be dependent on the presence of different pro-fibrotic cytokines rather than on TGFβ1. Interestingly, in the group treated with IFNγ and PPB-PEG-IFNγ, we noticed a trend towards reduction of PDGFRβ expression, which might explain their anti-fibrotic effects via downregulation of the PDGF/PDGFRβ signaling pathway. We observed that both IFNγ and PPB-PEG-IFNγ exert anti-fibrotic effects as they significantly dampened TGFβ-induced fibronectin, collagen I and collagen III expression after 48 h incubation. These observations confirmed our recent *in vivo* data (Poosti et al., 2015). However, in contrast to these *in vivo* data, in the current study PPB-PEG-IFNγ did not show higher efficacy when compared with free IFNγ. This could be explained by the fact that TGFβ1 did not increase PDGFRβ expression on mPCKS as described above. Besides, there is usually no added benefit of targeted compounds *in vitro*, as compounds can easily and effectively access the target cells, which is not the case after *in vivo* administration. Similar results were also obtained after exposure of NIH3T3 fibroblasts to IFNγ and PPB-PEG-IFNγ in the presence of TGFβ1 (Poosti et al., 2015). In fact, an effect after receptor-mediated endocytosis (as for PPB-PEG-IFNγ) sometimes may even be slower than a pharmacological effect of compounds directly to its receptor. The *ex vivo* studies with PPB-PEG-IFNγ therefore demonstrate that the targeted construct is pharmacologically active and was included as proof of concept.

To determine whether the anti-fibrotic effects were not due to PPB-induced blockade of PDGFRβ-signaling, we coupled PPB to albumin (HSA). We did not observe remarkable anti-fibrotic effects of PPB-HSA *ex vivo*; only on the mRNA level a (non-significant) effect of PPB-HSA on collagen III expression was observed, indicating that most of the observed anti-fibrotic effects are IFNγ-mediated. Biological effects of IFNγ take place via the nuclear signaling sequence (NLS) present in the C-terminus region of IFNγ. IFNγ containing NLS is capable to bind to IFNγ receptor1 (IFNγR1) and initiate a cascade of events required for nuclear import of STAT1 and generation of biological activity. Activation of STAT1 by PEG-PPB-IFNγ was previously shown (Bansal et al., 2011a). We propose that PEG-PPB-IFNγ is taken up via PDGFRβ and the internalized construct next releases IFNγ or its metabolite, which then binds to the intracellular part of IFNγR1 and activates the JAK/STAT pathway. However, this premise needs to be further explored.

**Fig. 6.**
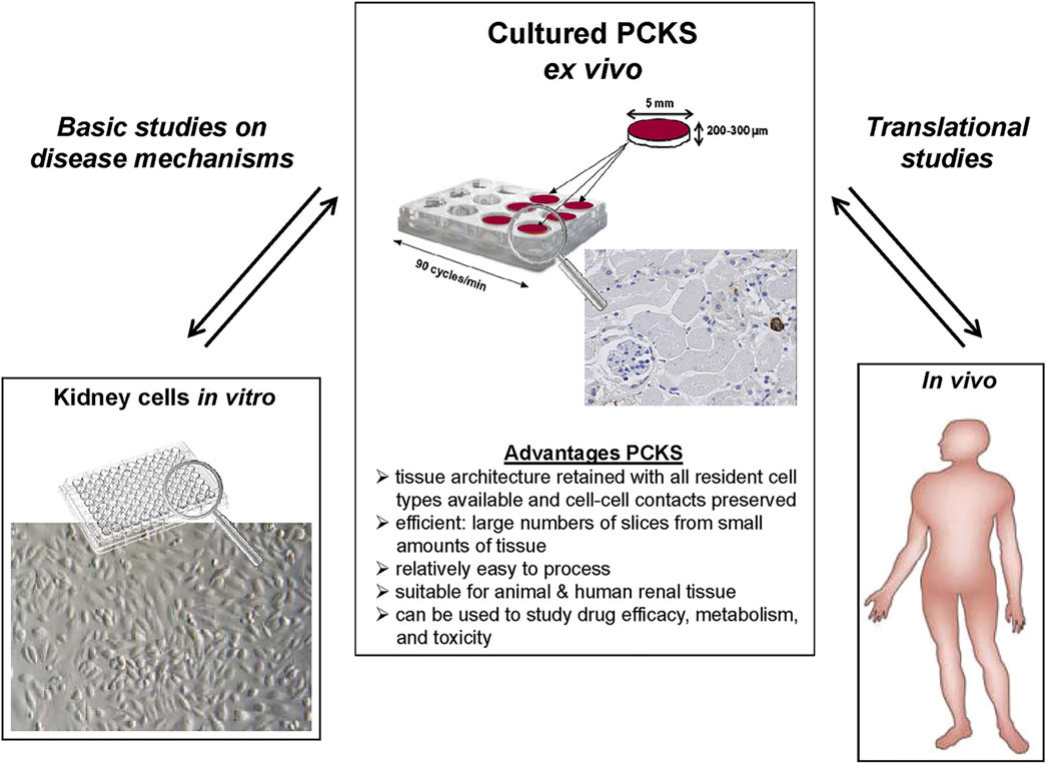
**The *ex vivo* PCKS model bridges the gap between basic research performed in *in vitro* cell culture models and translational *in vivo* human studies on pathophysiology and therapy of renal disease.** *In vitro* cultured cells displayed are primary human tubular epithelial cells (PTECs).

In summary, the mPCKS model is a novel tool to study the pathophysiology of early fibrotic processes not only in animal tissue, but also in (fibrotic) human kidney tissue. Importantly, from the results of this study we conclude that the *in vivo* observed anti-fibrotic effect of IFNγ and PPB-PEG-IFNγ can be successfully reproduced to great extent using mPCKS. This indicates that this *ex vivo* model is a useful tool for preclinical studies to test the efficacy of potential new anti-fibrotic drugs on fibroblast activation in a multicellular, pro-fibrotic milieu. In addition, the use of this model can contribute substantially to the reduction, refinement and potential replacement of animal experiments (the ‘3R’ principles). Further studies are ongoing to investigate the application of non-fibrotic and fibrotic human PCKS in order to validate our targeting strategy in the human setting. We believe that the PCKS model is able to bridge the gap between basic research performed using *in vitro* cell culture systems and translational human *in vivo* studies, as schematically represented in Fig. 6. The preclinical studies using PCKS could then pave the road towards clinical studies on cell-specific targeting of renal fibrosis.

## MATERIALS AND METHODS

### Mice

Male C57BL/6 mice weighing 25-30 g were obtained from Harlan (Zeist, The Netherlands). Animals were housed in individual cages with free access to food and water. Before starting the experiment, animals were allowed to acclimatize for at least 7 days. The experimental protocol was approved by the Animal Ethical Committee of the University of Groningen (DEC 6427A).

### Excision and preparation of mouse kidney slices

**Fig. 7.**
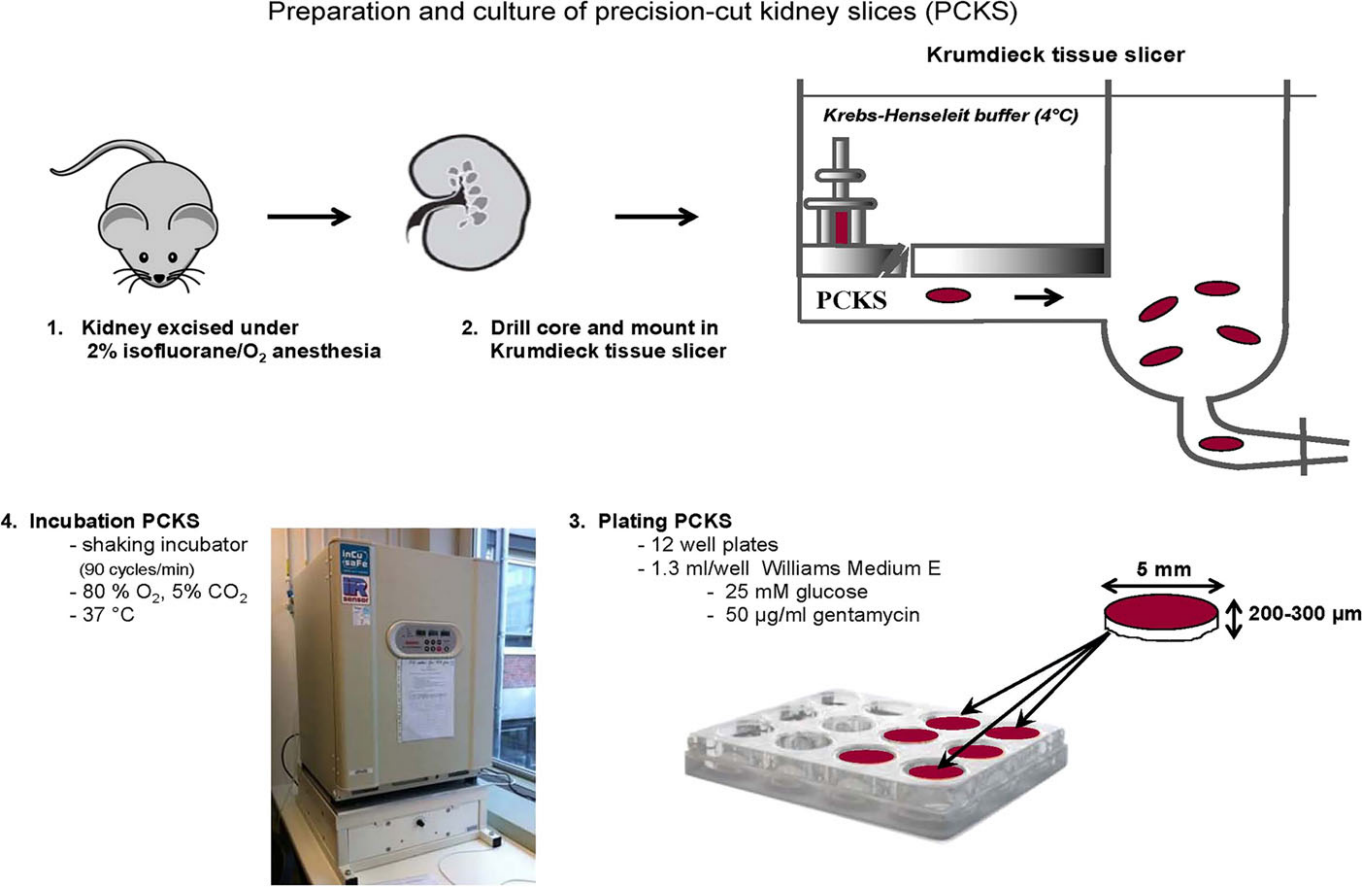
**Schematic representation of the experimental approach for obtaining and culturing mPCKS.** (1) Mouse kidneys (*n*=3) were excised under isofluorane/O_2_ anesthesia and placed into ice-cold UW preservation solution. (2) Cores were drilled and immediately transferred to the cylindrical core holder of a Krumdieck slicer and cut into 5-mm diameter slices. (3) High-quality slices (equal thickness at all sides and smooth edges) were transferred to 12-well plates using a spatula to avoid damaging of the slices, and (4) were placed in a shaking (90 cycles/min) incubator under continuous supply of 80% O_2_/5% CO_2_ at 37°C.

Slices were cultured in the presence or absence of TGFβ1 as described below. The protocol of the preparation and culture of kidney slices is illustrated in Fig. 7, and a detailed description is given below.

#### Preparation of Krebs–Henseleit buffer (KHB)

KHB was used as slicing buffer, and 10 l of a 10× concentrated KHB stock solution (10× KHB) were prepared by first dissolving 36.7 g CaCl_2_·2H_2_O in 5 l of ultrapure water (solution 1). Then, 37.3 g KCl, 690 g NaCl, 27.1 g MgSO_4_·7H_2_O and 16.3 g KH_2_PO_4_ were dissolved in ultrapure water to a volume of 5 l (solution 2). Subsequently, solutions 1 and 2 were mixed and filtered through a 0.45-µm filter. This 10× KHB can be stored at 4°C for about 6 months. On the day of slicing, 5 l 1× KHB were prepared by dissolving 10.5 g NaHCO_3_ (Merck, Darmstadt, Germany), 24.75 g D-glucose monohydrate (Merck, Darmstadt, Germany) and 11.9 g HEPES (MP Biomedicals, Aurora, OH, USA) in ∼2 l of ultrapure water at 4°C. Then, 500 ml 10× KHB and ultrapure water at 4 °C were added to yield a final volume of 5 l. This solution was kept at 0-4°C on melting ice, and was oxygenated with 95% O_2_/5% CO_2_ for 30 min on ice, using a gas dispersion tube with a fritted disc. The pH of the solution was adjusted to 7.42 by slowly adding 5N NaOH solution. This solution can be stored for 24 h at 4°C. Before use, the buffer was reoxygenated and the pH was readjusted.

#### Williams medium E glutamax-I (WME) slice incubation medium

On the day of the experiment, slice incubation medium was prepared by adding 1.375 g D-glucose monohydrate (Merck, Darmstadt, Germany) and 500 µl gentamicin (50 mg/ml) (Invitrogen, Paisley, UK) to 500 ml Williams medium E with GlutaMAX (Invitrogen, Paisley, UK). If necessary, this solution can be stored for 24 h at 4°C.

#### Slicing procedure

##### Preparations before start of slicing

KHB for slicing and WME (plus supplements) incubation medium were prepared as described above. WME incubation medium was transferred into culture plates, with 12-well plates requiring 1.3 ml of culture medium per well to support the tissue for 24 h. The volume-to-surface ratio of the culture medium is of great importance for optimal exchange of O_2_ and CO_2_ between the culture medium and the atmosphere of 80% O_2_/5% CO_2_, and the ratio of tissue to volume is important for sufficient supply of nutrients. The plates were pre-warmed and oxygenated by placing them in the shaking incubator at 37°C under 80% 80% O_2_/5% CO_2_ for at least 30 min. The Krumdieck slicer (Alabama Research and Development, USA) was assembled according to the manufacturer's instructions and pre-cooled by recirculating cooled water (4°C) through the cooling block, using a refrigerated circulator bath.

##### Collection of kidney tissue

Mice were anesthetized under 2% isofluorane/O_2_ (Nicholas Piramal, London, UK), the right and left kidneys were retrieved as quickly as possible and placed into ice-cold University of Wisconsin (UW) organ preservation solution (DuPont Critical Care, Waukegab, IL, USA). All further steps up to incubation were performed on ice (at 0-4°C). The mouse kidneys were transferred to a Petri dish with a silicone insert in order to remove adipose tissue around the kidney. The whole kidney was then placed into the core cylinder.

##### Preparation of kidney slices

The slicer was filled with ice-cold oxygenated KHB through the glass-trap assembly. It was ensured that the buffer reached the level required for proper functioning of the pump and that the tissue core was completely submerged. For mouse kidney slices, whole kidneys were transferred from the ice-cold UW solution used for storage into the cylindrical core holder of the slicer.

Slices were cut using the Krumdieck slicer at a speed setting of 30-40. The wet weight of the first few slices was checked as an indication of slice thickness after carefully blotting the slices on a smooth, not too absorptive paper to remove adherent water, with an optimal wet weight of 5 mg.

If necessary, the cutting thickness was adjusted by turning the graduated thickness control knob until the required wet weight was reached. The slice thickness can be slightly increased by placing weights on top of the tissue core holder, which may also facilitate the slicing of hard (fibrotic) kidney tissue. Kidney slices that are too thick (>400 μm) may develop necrosis in the center of the slice, as the diffusion distance for nutrients and oxygen is too long. After slicing one core, the slices were removed from the glass trap by opening the tap, collecting them in a beaker and placing them immediately on ice. Slices were selected on the basis of their appearance, with good slices having an equal thickness, uniform color and smooth edges. Selected slices were transferred into fresh ice-cold UW and stored on ice.

A spatula (not forceps) was used to avoid damaging the slices. The KHB slicing buffer was replaced by fresh, ice-cold and oxygenated KHB every 15-30 min, and the knife was replaced when slice quality (judged by eye) decreased.

### Experimental setup slice cultures

In this study, three independent experiments were performed, each using four kidneys (two mice), from which in total four cores were drilled. After slicing, slices were pooled before plating them for culture. ATP measurements were performed on single slices. For mRNA expression analysis, in each experiment slices were cultured in triplicate for each condition, after which they were pooled for RNA isolation. Histological analyses were performed on single slices per condition in each experiment.

### Incubation of kidney slices

Kidney slices were washed quickly by gently transferring them into WME to remove the UW. Before adding medium and mPCKS, 12-well plates were coated with 10% bovine serum albumin (BSA) in milliQ water for 30 min. The solution was removed and plates were air-dried. Slices were then incubated in the coated plates containing 1.3 ml Williams Medium E glutamax-I (Gibco, Paisley, UK) per well, supplemented with 25 mM D-glucose and 50 µg/ml gentamycin. Medium was pre-warmed and gassed with 80% O_2_/5% CO_2_ before it was added to the wells. Slices were individually transferred to the wells of a culture plate placed on a surgical mattress to maintain the medium at 37°C. Plates were immediately transferred back in a shaking CO_2_ incubator (90 cycles/min) under continuous supply of 80% O_2_/5% CO_2_, as the pH of the medium rapidly changes in normal atmosphere. Slices were incubated for 1, 6, 12, 24, 48 or 72 h. When appropriate, after 24 and 48 h slices were transferred to new plates with fresh medium. For refreshing, new culture plates were filled with medium, and the plates were pre-warmed and oxygenated. The incubated plates and new plates were placed on a surgical mattress to maintain the temperature at 37°C. The slices were quickly transferred from the incubated plates into the new plates with a spatula and placed in the incubator.

### Uptake of FITC-lysozyme by mPCKS

FITC-lysozyme was prepared as described before (Kok et al., 1998). Briefly, 500 µl of 1 mg/ml FITC (Thermo Scientific, Wilmington, MA, USA) was carefully mixed with 5 ml of 2 mg/ml lysozyme solution (Sigma-Aldrich, St Louis, USA) for 8 h at 4°C in the dark, dialyzed against water and finally lyophilized. Mouse kidney slices were incubated up to 3 h with 1 mg/ml FITC-lysozyme. After 1 and 3 h, the mPCKS were embedded in Tissue-Tek (Sakura, Japan) and snap-frozen in isopentane (−80°C). They were stored at −80°C until 4-μm cryosections were cut perpendicular to the surface of the slices. Cross-sections were allowed to dry on a glass microscope slide and covered with Citifluor (Citifluor, London, UK). Slides were examined using a high-end, fully motorized Zeiss AxioObserver Z1 microscope, and images were acquired using TissueFAXS Image Analysis Software (TissueGnostics, Austria). Images were converted to red/green pseudo-colors using lookup tables in ImageJ 1.47v (http://imagej.nih.gov/ij).

### Induction of renal pre-fibrosis *ex vivo*

In order to induce renal pre-fibrosis *ex vivo*, slices were incubated for 48 h in the presence of recombinant human TGFβ1 (5 ng/ml, hTGFβ1, Roche, Mannheim, Germany). Medium without TGFβ1 was used for comparison. After 24 h of incubation, slices were transferred to new coated plates with fresh medium containing TGFβ1.

### Synthesis of conjugates

PPB-PEG-IFNγ (in which PPB is the PDGFR-recognizing, binding peptide) was synthesized as described previously (Bansal et al., 2011a). Briefly, for PPB-PEG-IFNγ, recombinant murine IFNγ (0.256 nmol, Peprotech, London, UK) was mixed with 12.8 nmol of maleimide-PEG-succinimidyl carboxy methyl ester (Mal-PEG-SCM, 2 kDa, Creative PEGworks, Winston-Salem, NC, USA) for 2 h and dialyzed overnight against PBS using 10 kDa dispodialyzer (Harvard Apparatus, Holliston, MA, USA). The dialyzed sample was then mixed with SATA (succinimidyl acetylthioacetate)-modified PPB (PPB-ATA, 25.6 nmol) in the presence of a deacetylating reagent. Finally, PPB-PEG-IFNγ was extensively dialyzed against PBS and stored at −80°C.

For PPB-HSA, human serum albumin (HSA, 1.5 µmol dissolved in PBS) was mixed with γ-maleimidobutyryloxy-succinimide ester (GMBS, 30 µmol, dissolved in DMF) for 2 h and extensively dialyzed against PBS using 10 kDa cut-off dialysis membrane cassette (Thermo Scientific, Rockford, IL, USA). Next, PPB-ATA (34.5 µmol; dissolved in DMF) was added to the GMBS-modified HSA for overnight, and dialyzed against PBS. The final product (PPB-HSA) was freeze-dried for storage at −20°C.

### Anti-fibrotic effects of free IFNγ and PPB-PEG-IFNγ

To determine the anti-fibrotic effects of free IFNγ and PPB-PEG-IFNγ, slices were incubated for 48 h with free IFNγ, PPB-PEG-IFNγ, PPB-HSA (equivalent 1 µg/ml) or medium alone in the presence or absence of TGFβ1 as described above. After the first 24 h of incubation slices were transferred to new 12 well plates with fresh medium containing TGFβ1 and the respective anti-fibrotic compounds. TGFβ1 and IFNγ constructs were administered simultaneously. The concentration of 1 µg/ml of IFNγ constructs was chosen based on results from previous *in vitro* experiments (Bansal et al., 2011a; Poosti et al., 2015).

### Viability of mPKCS: ATP and protein content

The general viability of the PCKS during culture was determined by measuring ATP content of the slices. For ATP measurements, single slices were transferred to a sonication solution containing 70% ethanol (v/v) and 2 mM EDTA (pH 10.9), snap-frozen in liquid nitrogen and stored at −80°C until analysis. After homogenization using a Mini-BeadBeater-8 (BioSpec, Bartlesville, OK, USA), the samples were centrifuged for 2 min at 16,000 ***g***. The supernatant was diluted ten times with 0.1 M Tris-HCl/2 mM EDTA buffer (pH 7.8) to lower the ethanol concentration and was used to measure ATP contents using the ATP Bioluminescence assay kit CLSII (Roche Diagnostics, Mannheim, Germany) according to the manufacturer's protocol. The remaining pellet was used to determine the protein content of the mPCKS by dissolving it in 200 μl of 5 M NaOH for 30 min. After dilution with water to a concentration of 1 M NaOH, the protein content of the samples was determined using the Lowry method (Bio-Rad DC Protein Assay, Bio-Rad, Munich, Germany). BSA was used for the calibration curve. ATP values (in pmol) were divided by the total protein content (in μg) of the respective slices and expressed as the ratio ATP/protein.

### Quantitative real-time PCR

In order to determine TGFβ-induced pre-fibrosis, and to examine the effect of IFNγ and PPB-PEG-IFNγ, gene expression of fibrosis markers [alpha smooth muscle actin (α-SMA), fibronectin (Fn), collagens I and III] was measured using quantitative real-time (qRT)-PCR. The triplicate slices were pooled and snap-frozen. Total RNA from kidney slices was extracted using the RNeasy Mini Kit (Qiagen, Hilden, Germany) according to the manufacturer's instructions. The RNA concentrations were measured on a NanoDrop ND-1000 spectrophotometer (Thermo Scientific, Wilmington, MA, USA). Single-stranded cDNA was synthesized using Superscript II and random hexamer primers (Invitrogen, Carlsbad, CA) in a volume of 20 µl. cDNA was first diluted to a concentration of 2 ng/μl and 2.5 μl/reaction (5 ng), and was then used for qRT-PCR analysis. PCR reactions were performed in a 10-µl reaction volume containing 1× qPCR master mix (Eurogentec, Liege, Belgium) and 1× Taqman Gene Expression Assay mix (Applied Biosystems, Foster City, CA, USA). The Taqman assay numbers were as follows: Ywhaz: Mm03950126_s1, Col1a1: Mm00801666_g1, Col3a1: Mm01254476_m1, α-SMA/Acta2: Mm01546133_m1, Fn: Mm01256744_m1, MHCII/CD74: Mm00658576_m1. qRT-PCR reactions were performed on a ABI7900HT thermal cycler (Applied Biosystems, Foster City, CA, USA). Relative gene expression was calculated using the 2^−ΔΔCt^ method, with *Ywhaz* (tyrosine 3-monooxygenase/tryptophan 5-monooxygenase activation protein ζ) as housekeeping gene.

### Histology of mPKCS

Study of histological changes was performed on 2-µm sections from formalin-fixed paraffin-embedded slices. For immunohistochemistry the following primary antibodies were used: anti-alpha smooth muscle actin (α-SMA, clone ASM-1, Progen Biotechnik, Heidelberg, Germany); anti-fibronectin (ab6584, Abcam, Cambridge, UK); and anti-collagen III (s1330-01, SouthernBiotech, Birmingham, AL, USA). Sections were deparaffinized in xylene and rehydrated in graded alcohol and distilled water. Antigen retrieval was achieved by overnight incubation at 60°C in 0.1 M Tris/HCl buffer (pH 9.0) for fibronectin and collagen III staining. No antigen retrieval was performed for α-SMA staining. Endogenous peroxidase activity was blocked with 0.03% H_2_O_2_ (in PBS) for 30 min. Primary antibody binding was detected by sequential incubations with horseradish peroxidase (HRP)-labeled appropriate secondary and tertiary antibodies (obtained from DAKO, Glostrup, Denmark). Peroxidase activity was visualized using 3,3′-diaminobenzidine tetrahydrochloride (DAKO, Glostrup, Denmark) as chromogen (10 min incubation). Sections were counterstained with hematoxylin for 1 min and mounted with Kaiser's glycerin gelatin.

### Quantification of immunostaining

To quantify immunostaining of fibronectin and collagen III, sections were first scanned using a NanoZoomer HT (Hamamatsu Photonics K.K., Shizuoka Pref., Japan). Next, the extent of fibronectin and collagen III^+^ staining was measured (number of positive pixels) using Aperio ImageScope software (version 9.1.772.1570, Aperio Technologies, Vista, CA, USA).

### Statistical analyses

Statistical analyses were performed using GraphPad Prism 5.0 (GraphPad Software, La Jolla, CA, USA). Data are expressed as mean±s.e.m. of three independent experiments. Differences between multiple groups were calculated using ANOVA. Comparisons of two groups were performed using Student's *t*-test. *P*<0.05 was considered statistically significant.

## Notes

This article is part of a subject collection on Model Systems in Drug Discovery: from Bench to Patient. See related articles in this collection at http://dmm.biologists.org/cgi/collection/drugdiscovery
